# Assessment of the Radiographic Angular Parameters in the Coronal Plane of the Lower Limbs in Subjects without Knee Osteoarthritis in a Reference Hospital of the Brazilian Unified Health System

**DOI:** 10.1055/s-0044-1792118

**Published:** 2024-12-21

**Authors:** Diego Perez da Motta, Larissa da Silva, Leandro Lemgruber Kropf, Fernando dos Santos Cerqueira, Bruno Domenico Leonetti, Flavio dos Santos Cerqueira

**Affiliations:** 1 Centro de Atenção Especializada do Tratamento da Dismetria e Deformidades do Aparelho Locomotor, Instituto Nacional de Traumatologia e Ortopedia, Rio de Janeiro, RJ, Brasil; 2 Programa de Residência Médica, Instituto Nacional de Traumatologia e Ortopedia, Rio de Janeiro, RJ, Brasil

**Keywords:** genu valgum, genu varum, joint deformities, acquired, limb deformities, congenital, osteoarthritis

## Abstract

**Objective** The present study aims to demonstrate the radiological angular parameters of a sample of patients treated at our institution and to compare the radiological abnormalities with other classifications or parameters from the literature.

**Methods** We evaluated a sample of patients submitted to panoramic radiographic examinations of the lower limbs. The inclusion criteria were: (1) Patients without knee osteoarthritis as assessed by an orthopedist. (2) Bilateral radiographic evaluation in the panoramic examination of the lower limbs. (3) Panoramic radiographic examination of the lower limbs before any surgical procedure. (4) Patients over 18 years old.

**Results** We analyzed a total of 1,242 lower limbs. The axis was neutral in 875 lower limbs (70.4%). The main etiology was non-traumatic, with a varus deviation of the tibial segment in 253 cases and the deformity apex in the proximal third of the tibial segment. A valgus deviation of the mechanical axis was more common in non-traumatic etiologies (82.3%). Per the Coronal Plane Alignment of the Knee (CPAK) classification, type I was the most frequent (44.8%), followed by type III (37.1%).

**Conclusion** We identified changes in the angular parameters in patients from a reference hospital from the Brazilian Unified Health System which were different from population samples from other countries.

## Introduction

Changes in the mechanical axis of the lower limbs are frequent reasons for treatment in different orthopedic specialties. Planning for correcting these deviations relies on a thorough clinical evaluation and radiological assessment. 1 For the physical examination, a visual evaluation of the lower limb axis is critical, as it provides the evaluator with probable bone deviation and allows the visualization of soft tissue conditions that may turn any surgical procedure unfeasible. 2 The radiographic examination must be a panoramic radiograph of the lower limbs in the orthostatic position to trace the mechanical and anatomical axes and the joint angles to assess angular parameters, determine the presence of deformities (if in the femoral, tibial, or both segments), and plan the correction. 3 4 5

**Fig. 1:**
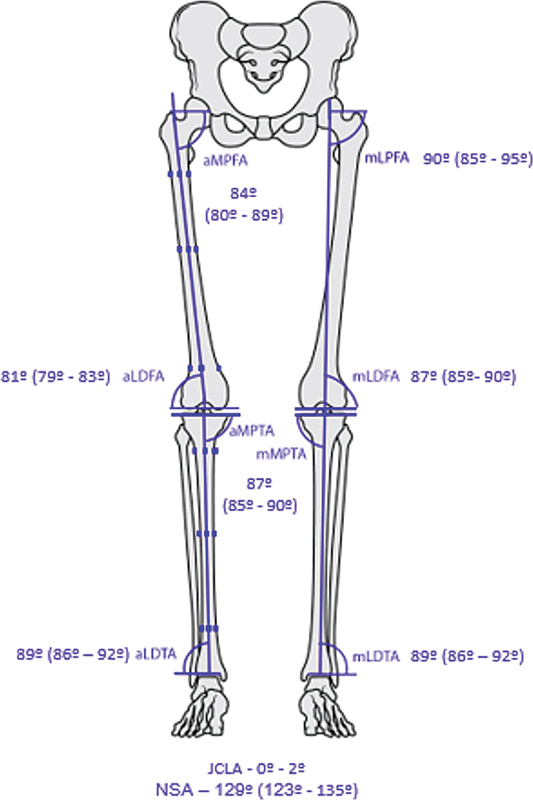
Schematic representation of the relationship between angles and the anatomical and mechanical axes and their reference values. Abbreviations: aLDFA, anatomical lateral distal femoral angle; aLDTA, anatomical lateral distal tibia angle; aMPFA, anatomical medial proximal femoral angle; aMPTA, anatomical medial proximal tibia angle; JCLA, joint line convergence angle; mLDFA, mechanical lateral distal femoral angle; mLPFA, mechanical lateral proximal femoral angle; mLDTA, mechanical lateral distal tibia angle; mMPTA, mechanical medial proximal tibia angle; NSA, Neck-shaft angle;.

Paley et al. 6 7 8 standardized the angle nomenclature to optimize communication and outcome comparison. Additionally, they organized the planning of the deformity correction by segment. The angle nomenclature consists of letters referring to the axis type (anatomical or mechanical), whether the angle is medial or lateral, its location (femur or tibia), and segment position (proximal or distal). Additionally, joint orientation lines, such as those of the femoral condyles and tibial plateaus, generate the joint convergence line angle (JCLA), ranging from 0 to 2 degrees, and the neck-shaft angle (NSA), with a variation of 129 ± 6 degrees. This planning describes the methodology for evaluating the deformity apex (center of rotation of angulation, CORA) using the intersection between the angles in the evaluated segment ( Fig. 1 ). 6 7 8

Deformity etiologies can belong to two general groups: traumatic or non-traumatic. Traumatic deformities originate from conservative or surgical fracture treatment. Non-traumatic deformities result from genetic growth defects. 9 10 11 12 The mechanical axis of the lower limbs is neutral when it does not present a deviation from the midpoint of the knee. An axis deviation to the medial or lateral side from this midpoint determines that the mechanical axis of the lower limb is varus or valgus, respectively. 13 14 15

In the literature, the classifications rely on assessing knee joint wear and demonstrate specific radiographic patterns in populations other than the Brazilian one. 16 17 18 No study described the general radiographic patterns in Brazilian patients. This study aimed to describe the angular parameters of the lower limbs in patients treated at our institution and compare them with those described in the literature.

## Materials and Methods

The Research Ethics Committee approved the present retrospective cross-sectional study under number CAAE 68507323.8.0000.5273 with patients who underwent radiographic evaluation at our hospital from January 2012 to July 2023.

The inclusion criteria were: (1) Patients without knee osteoarthritis as assessed by an orthopedist. (2) Bilateral radiographic evaluation in the panoramic examination of the lower limbs. (3) Panoramic radiographic examination of the lower limbs before any surgical procedure. (4) Patients over 18 years old.

The exclusion criteria were: (1) Patients who underwent a surgical procedure to correct a deformity before the radiographic examination at any hospital. (2) Patients with rotational changes in the lower limb or inability to measure them during the radiographic examination, hindering its quality (3). Presence of osteoarthritis in the radiographic examination. (4) Lack of information in the physical medical record.

Our institutional radiology database provided the radiographic examinations. We used the Peekmed software (PeekMed LLC, Braga, Portugal) to measure the angular parameters. We entered these data in a Microsoft Excel (Microsoft Corp., Redmond, WA, USA) spreadsheet with no identification by medical record number or initial name, employing only codes registered by the main researcher for later statistical evaluation. The only variables collected from the physical medical records were age and gender.

The total sample classification followed the parameters suggested by MacDessi et al., 16 that is, the Coronal Plane Alignment of the Knee (CPAK) Classification. This classification has nine phenotypes according to the relationship between the joint line obliquity (the sum of the medial proximal tibia angle and the lateral distal femoral angle) and the arithmetic difference between these angles, which shows the axis type. A negative difference indicates a varus axis; a difference equal to zero, a neutral axis; and a positive difference, a valgus axis. The joint line obliquity only informs the site of the obliquity's apex.

## Statistical Analysis

The analyses were performed descriptively for quantitative data and presented as mean, standard deviation, median, minimum, and maximum values. We expressed categorical variables as frequencies and percentages.

We used the JASP software, version 0.18 (open source) to compare parameters with a normal distribution using the Student t-test. To analyze categorical variables, we employed the Chi-squared test, considering a *p* -value < 0.05 as significant.

## Results

We detected 621 patients (1,242 lower limbs) who underwent radiographic examinations from January 2012 to July 2023.

Of the total sample, 498 subjects were male (80.1%) and 123 were female (19.9%). The mean age of the sample was 33.4 years (standard deviation = 10.1). Comparing mechanical axis deviations with no evaluation of the deformed segment regarding absolute frequency, gender, and age, 875 limbs presented a neutral axis (686 limbs in males and 189 limbs in females). The mean age of these subjects was 32.9 years, ranging from 18 to 67 years. Groups with valgus or varus axis deviation were similar regarding mean age and range. Their mean age was 34.9 years, ranging from 18 to 66 years, totaling 367 lower limbs (310 male and 57 female limbs) ( Table 1 ).

**Table 1:** Descriptive measurements when comparing the mechanical axis deviations

	Group	N	Mean	Median	Standard deviation	*P* -value
**Age (years)**	Neutral	875	32.92	32.00	9.956	0.004
	Valgus/varus	367	34.75	34.00	10.65	
**NSA (degrees)**	Neutral	875	126.94	127.00	5.845	< 0.001
	Valgus/varus	367	128.78	128.00	5.24	
**LPFA (degrees)**	Neutral	875	90.57	90.00	4.639	< 0.001
	Valgus/varus	367	92.49	91.00	6.55	
**LDFA (degrees)**	Neutral	875	86.72	87.00	1.530	0.176
	Valgus/varus	367	86.52	86.00	3.75	
**JCLA (degrees)**	Neutral	875	1.32	1.20	0.685	< 0.001
	Valgus/varus	367	1.76	1.50	1.30	
**Size of the joint line obliquity(degrees)**	Neutral	875	1.44	1.10	1.062	< 0.001
	Valgus/varus	367	2.23	1.80	1.86	
**MPTA (degrees)**	Neutral	875	87.16	87.00	1.588	< 0.001
	Valgus/varus	367	84.27	83.00	4.20	
**LDTA (degrees)**	Neutral	875	88.04	88.00	1.458	< 0.001
	Valgus/varus	367	89.04	89.00	4.49	

Abbreviations: JCLA, Joint line convergence angle; LDFA, lateral distal femoral angle; LDTA, lateral distal tibia angle; LPFA, lateral proximal femoral angle; MPTA, medial proximal tibia angle; NSA, neck-shaft angle.

Note: Student t-test.

By assessing patients with a mechanical axis deviation in the lower limbs, without relating the deformed segment, we noted a predominance of varus over valgus deviation in 84.4% of the subjects. Bilateral deviations occurred in 108 patients (216 limbs) with varus and 15 subjects (30 limbs) with valgus. Varus was more frequent in males (56.4% of cases; however, valgus was more common in females [50.8% of patients]) ( Table 2 ).

**Table 2:** Angular patterns of limbs that deviated from the mechanical axis

	Axis type	N	Mean	Median	Standard deviation	Minimum	Maximum	*P* -value
Age (years)	Varus	310	35.55	32	10.14	18	67	< 0.001
	Valgus	57	31.48	32	10.12	18	56	
NSA (degrees)	Varus	310	128.83	128.50	5.23	115	143	0.686
	Valgus	57	128.53	127	5.30	120	150	
LPFA (degrees)	Varus	310	92.56	91.00	6.50	74	115	0.604
	Valgus	57	92.07	92	6.88	70	108	
LDTA (degrees)	Varus	310	87.15	87.00	3.08	73	107	< 0.001
	Valgus	57	83.11	85	5.05	72	97	
JCLA (degrees)	Varus	310	1.72	1.50	1.22	0.100	8.30	0.183
	Valgus	57	1.97	1.50	1.69	0.100	7.20	
Joint line obliquity (degrees)	Varus	310	2.03	1.70	1.36	0.200	9.00	< 0.001
	Valgus	57	3.29	2.00	3.32	0.200	19.00	
MPTA (degrees)	Varus	310	83.08	83.00	2.65	75	95	< 0.001
	Valgus	57	90.77	92	5.08	81	115	

Abbreviations: JCLA, joint convergence line angle; LDFA, lateral distal femoral angle; LDTA, lateral distal tibial angle; MPFA, medial proximal femoral angle; MPTA, mechanical proximal tibial angle; NSA, neck-shaft angle.

Note: Student t-test.

In patients with a non-traumatic etiology for mechanical axis deviation, varus was more frequent in isolated deformities in the tibial segment, affecting 253 limbs. In the femoral segment, 10 limbs presented isolated deformities. Nevertheless, concomitant deformities in the tibial and femoral segments occurred in 17 limbs.

In patients with a traumatic etiology, there was a predominance of isolated varus in the tibial (10 limbs) and the femoral (8 limbs) segments. Concomitant varus occurred in two limbs. Ten limbs had a concomitant deformity with different etiologies in different segments.

Regarding the deformity apex, in cases of non-traumatic etiology, the most frequent location was the proximal third in the tibial varus deformity. In traumatic etiologies, the highest frequency was in the middle third in the femoral varus deformity.

In the valgus mechanical axis deviation, isolated tibial deformities resulted from both etiologies (7 limbs with traumatic etiology and 32 limbs with non-traumatic etiology). In the femur, the non-traumatic etiology was present in seven cases and the traumatic etiology in one case. In deformities affecting both segments (femur, and tibia), 10 cases resulted from non-traumatic etiology. The deformity location was predominantly in the middle third of the tibia in both non-traumatic and traumatic etiologies ( Table 3 ).

**Table 3:** Description of the type of mechanical axis deviation according to the etiology, location and angular variation of the sample

Axis type	Etiology	Deformity location	Deformity size (degrees) (minimum–maximum; mean)
**Varus**	**Non-traumatic**	Medial tibia	6 **–** 14; 9
		Proximal tibia	2 **–** 14; 8
		Medial femur	4–22; 12
		Distal femur	3–30; 9
	**Traumatic**	Medial tibia	8–20; 9
		Tíbia distal	7–21; 14
		Proximal tibia	7–20; 13
**Valgus**	**Non-traumatic**	Distal femur	3–20; 8
		Medial femur	26
		Proximal tibia	3–21; 7
		Medial tibia	3–10; 6
		Distal tibia	4–19, 12
	**Traumatic**	Medial femur	5
		Medial tibia	13–26; 19

In the anatomical knee region, in addition to the mechanical axis-related joint angles, we can trace angles between the articular surfaces of the femoral condyles and tibial plateaus (JCLA) and between this articular line of the tibial plateaus and the horizontal line on the ground (obliquity type of the articular line of the tibial plateaus).

The sample with valgus deformity of the mechanical axis of the limb presented proportionally more cases of JCLA abnormalities and obliquity ranges compared with varus deformity ( Table 4 ).

**Table 4:** Variation in the joint line convergence angle (JCLA), and obliquity type and range parameters in the sample's knees

JLCA range	Valgus	Varus	Neutral
0–2	35	223	796
2.1–5	20	81	78
> 5.1	3	5	1
**Obliquity type**			
**Lateral tilt (LT)**	26	259	675
**Medial tilt (MT)**	32	50	200
**Obliquity range**			
0–2	30	185	726
2.1–4	13	104	122
4.1–6	8	15	23
6.1–8	1	4	3
> 8	6	1	1

**Fig. 2:**
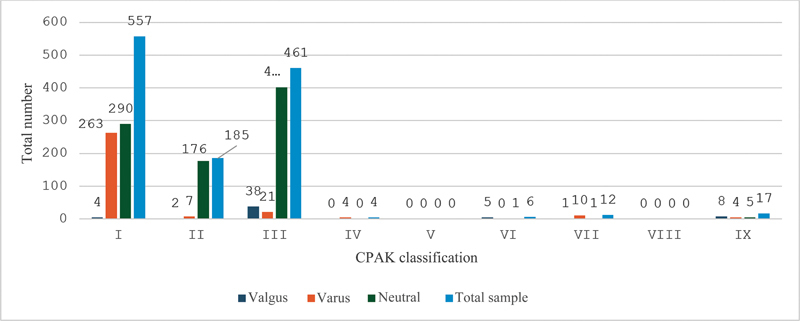
Sample classification according to the Coronal Plane Alignment of the Knee (CPAK).

The total sample and respective groups underwent the CPAK classification according to the parameters suggested by MacDessi et al. 16 In the total sample, type I of the CPAK classification was the most frequent (44.8%), followed by type III (37.1%). The varus sample followed the same pattern as the general sample. However, in the valgus sample, type III was more frequent than type I ( Fig. 2 ).

## Discussion

Deformities require correction due to the increased pressure in the knee compartments, which alters chondrocyte homeostasis and accelerates the osteoarthritis process. 19 20 21 22 With the so-called neutral axis, the subject's weight overloads the medial compartment by up to 60%. In lateral deviations of the mechanical axis, valgus occurs; for each degree of lateral deviation of the weight force, there is an increased chance of unicompartmental osteoarthritis by up to 55% and severe osteoarthritis by 19% in the 3 knee compartments. 20 23 However, early joint wear does not occur in the knee alone. Increased overload also occurs in the ankle joints and the foot joint complex, generating compensatory changes that allow the subject to walk with the lowest energy and joint expenditure. 24 25 The neutral mechanical axis, with no segmental deformities, presents the parallelism of the lower limb joints to the ground.

The current literature has no classification relating etiology to the affected segment and demonstrating the most common patterns in each deformity. Mullaji et al. 18 described a classification for phenotypes with valgus deformity of the lower limb to guide the balance of soft tissues during total knee arthroplasty, with no mention of other deformities and etiologies. MacDessi et al. 16 presented another classification based on the morphology of the tibial plateau articular line and the deviation type of the mechanical axis but did not report the CORA location and its magnitude.

Our study demonstrated a predominance of varus deviation of the mechanical axis, consistent with the findings of Moon et al. 26 and Krajnc and Drobnič. 17 However, these studies are different from ours; for instance, the study by Krajnc and Drobnič had younger patients (64.6 ± 13 years), and Moon et al. used 94 panoramic radiographs of the lower limbs with patients who could have knee osteoarthritis. Degenerative processes in the knee joint can overestimate the values of the angular parameters. Additionally, there is a lack of discriminated angular parameters in the study by Moon et al., hindering a complete comparison. 26 Krajnc and Drobnič 17 evaluated the angular parameters of 48 professional soccer players with a mean age lower than that of our study (24.2 ± 3.6 years), and 87.5% of their subjects had varus deviation of the mechanical axis of the lower limbs. When comparing the sample treated in a hospital with professional athletes, the angular parameters were similar to those proposed by Paley et al. 8 and us. Despite the similarity of the angular parameters and deviation type of the mechanical axis with our study, there is no mention of knee joint line obliquity, JCLA, and NSA.

Classifying the mechanical axis type of the lower limb using panoramic radiographs is easy. In an ideal situation, there would be collinearity between the center of the femoral head, the knee, and the ankle. The joints of the lower limbs and their joint reference lines must parallel each other and the ground, with the axis passing at the center of the knee and ankle (theory of joint parallelism). Nevertheless, segmental assessment is complex due to the possibility of complex deformities in one or both segments that, when added, result in mechanical axis deviation. The phenotype of the Brazilian population is diverse and difficult to standardize. The pattern presented by our patients differs from the sample of subjects without osteoarthritis evaluated by MacDessi et al. 16 MacDessi et al. evaluated 2 groups of Australian patients, one consisting of healthy people with no orthopedic complaints aged 20 to 27 and another with no knee osteoarthritis and classified per the axis and obliquity type of the knee joint line (CPAK classification). The most frequent type was type II of the CPAK classification, followed by type I, in contrast to our results. 16 Our study used radiographic examinations of patients presenting some orthopedic complaints, leading to the need for the examination. This may explain the difference between our standard and the Australian sample, in addition to the difference in population phenotyping. Toyooka et al. 27 used the same classification to compare a sample of the Japanese population with osteoarthritis, with results consistent with those of our study and most subjects (53.7%) classified as type I.

The obliquity of the knee line and the JCLA presented a predominance within normal standards, consistent with Kubota et al. 28 These authors followed up 68 participants with pre- and postoperative evaluation after valgus osteotomy of the proximal tibia due to varus deviation of the mechanical axis and osteoarthritis of the medial compartment of the knee. In the preoperative assessment, the knee joint line obliquity was 1.1 ± 3.3 degrees and the JCLA was 3.4 ± 2.2 degrees, without identifying the CORA or deformity size. The significance of these joint parameters is the risk of early development of knee osteoarthritis, as cited by Song et al. 29 These authors evaluated 109 patients who underwent open medial tibial osteotomy from 2010 to 2015, with a mean follow-up of 55 months, to assess the knee line obliquity. When this angle was greater than 6 degrees, there was a positive relationship with knee osteoarthritis on the radiographic examination; if higher than 4 degrees, there was an increase in negative clinical outcomes.

Bagaria et al. 30 studied 2,279 patients with knee osteoarthritis from 1990 to 2019 with panoramic radiography of the lower limbs. They detected that the highest frequency of phenotypes was varus deviation of the mechanical axis (38.7%), followed by neutral (37%). However, the most frequent phenotype was femoral varus with a neutral tibia. Our study presented similar results regarding the type of deviation found in the population; however, unlike their findings, the neutral axis was the most frequent and, in the presence of deviation, varus was more common than valgus. In addition, the varus deformity was more frequent in the tibia than in the femur.

The present study had limitations regarding the definition of the sample, since it evaluated only patients over 18 years old without osteoarthritis. The radiographic evaluation had a significant loss of participants due to poor positioning for the panoramic radiography of the lower limbs. Comparison with other studies is difficult because there are no descriptive studies with all the angular parameters, only specific studies of each parameter, and no studies with a Brazilian population sample. The miscegenation of the Brazilian population can cause different phenotypes in different regions of the country, potentially leading to different results. Further studies with larger samples and in sites that are not reference hospitals in orthopedics are required to compare the results presented in this study and those described in the literature.

## Conclusion

We identified changes in the angular parameters presented by a sample of patients from a Brazilian Unified Health System reference hospital with characteristics different from population samples from other countries.
