# Supplementary material for: Assessment of the Radiographic Angular Parameters in the Coronal Plane of the Lower Limbs in Subjects without Knee Osteoarthritis in a Reference Hospital of the Brazilian Unified Health System
Source: Rev Bras Ortop (Sao Paulo). 2024 Dec 21;59(6):e839–46. doi: 10.1055/s-0044-1792118 (PMC11663054; doi:10.1055/s-0044-1792118)
Supplement: Related texts

# Research article: Avaliação dos parâmetros angulares radiográficos no plano coronal dos membros inferiores em indivíduos sem osteoartrite de joelho em um hospital de referência do Sistema Único de Saúde

**Keywords:** deformidades articulares adquiridas, deformidades congênitas dos membros, geno valgo, geno varo, osteoartrite

## Abstract

**Objetivo** Este estudo pretende demonstrar os parâmetros angulares radiológicos de uma amostra de pacientes atendidos em nossa instituição e comparar as alterações radiológicas com outras classificações ou parâmetros presentes na literatura.

**Métodos** Avaliar uma amostra de pacientes submetida ao exame radiográfico panorâmico de membros inferiores. Os critérios de inclusão utilizados foram: (1) Pacientes sem osteoartrite de joelho avaliados por ortopedista. (2) Avaliação radiográfica bilateral no exame panorâmico de membros inferiores. (3) Exame radiográfico panorâmico de membros inferiores realizado previamente a qualquer procedimento cirúrgico. (4) Pacientes com idade superior a 18 anos.

**Resultados** Foram avaliados 1.242 membros inferiores. O eixo neutro foi observado em 875 membros inferiores (70.4%) da amostra total. A etiologia não traumática foi a mais frequente observada, apresentando o desvio em varo do segmento tibial em 253 casos e localização do ápice da deformidade no terço proximal do segmento tibial. O desvio em valgo do eixo mecânico também foi observado mais comumente na etiologia não traumática (82.3%). Na comparação pela classificação *Coronal Plane Alignment of the Knee* (CPAK), o tipo I foi o mais frequente (44.8%), seguido pelo tipo III (37.1%) na amostra total.

**Conclusão** Foram identificadas alterações nos parâmetros angulares apresentadas pela amostra de pacientes de um hospital de referência do Sistema Único de Saúde (SUS) com características diferentes de amostras populacionais de outros países.

## Introdução

As alterações do eixo mecânico dos membros inferiores são causas frequentes de atendimento nas diferentes especialidades da ortopedia. O planejamento da correção do desvio do eixo mecânico dos membros inferiores é realizado com a avaliação clínica e seguido pela avaliação radiológica. 1 No exame físico, a avaliação visual do eixo dos membros inferiores é importante, pois fornece ao avaliador o provável desvio ósseo assim como a visualização das condições de partes moles que possam inviabilizar qualquer tipo de procedimento cirúrgico. 2 O exame radiográfico necessário é a radiografia panorâmica de membros inferiores em ortostase, na qual são traçados os eixos mecânico e anatômico assim como os ângulos articulares para avaliação dos parâmetros angulares, presença de deformidades (se no segmento femoral e/ou tibial) e planejamento da correção. 3 4 5

Paley et al. 6 7 8 padronizaram a nomenclatura dos ângulos para otimizar a comunicação e comparação dos resultados. Além de organizarem o planejamento da correção de deformidade por segmento. A nomenclatura dos ângulos é composta letras referentes ao tipo de eixo, anatômico ou mecânico; se o ângulo é medial ou lateral; qual o segmento em que ele se encontra, fêmur ou tíbia; e a posição neste segmento, proximal ou distal. Adicionalmente, podem ser traçadas as linhas de orientação articular como as dos côndilos femorais e platôs tibiais, que quando relacionadas geram o *joint convergence line angle* (JCLA), com variação de 0 a 2 graus; e o *neck shaft angle* (NSA), com variação de 129 ± 6 graus. Neste planejamento é descrita a metodologia de avaliação do ápice da deformidade ( *center of rotation of angulation - CORA* ) usando a intersecção entre os ângulos apresentados no segmento avaliado ( Fig. 1 ). 6 7 8

**Fig. 1:** Representação esquemática da relação dos ângulos com os eixos anatômico e mecânico e seus valores de referência. Abreviaturas: aLDFA, *anatomical lateral distal femoral angle* ; aLDTA, *anatomical lateral distal tibia angle;* aMPFA, *anatomical medial proximal femoral angle* ; aMPTA, *anatomical medial proximal tibia angle* ; JCLA, *joint line convergence angle* ; mLDFA, *mechanical lateral distal femoral angle* ; mLDTA, *mechanical lateral distal tibia angle* ; mLPFA, *mechanical lateral proximal femoral angle* ; mMPTA, *mechanical medial proximal tibia angle* ; NSA, *neck shaft angle* .

As etiologias das deformidades podem ser divididas em dois grupos de forma genérica: traumática ou não traumática. As traumáticas têm origem no tratamento conservador ou cirúrgico de fraturas. As não traumáticas têm origem nos defeitos genéticos do crescimento. 9 10 11 12 O eixo mecânico dos membros inferiores é dito neutro quando não apresenta desvio em relação ao ponto médio do joelho. Se houver desvio deste eixo para medial ou lateral em relação a este ponto médio, então é dito eixo mecânico do membro inferior em varo ou valgo, respectivamente. 13 14 15

As classificações existentes na literatura têm como cerne de avaliação o desgaste articular do joelho e demonstram padrões radiográficos específicos em populações diferentes da brasileira. 16 17 18 Não há estudo que descreva os padrões radiográficos gerais em um grupo de pacientes brasileiros. Este estudo pretende descrever os parâmetros angulares dos membros inferiores em pacientes atendidos em nossa instituição e comparar com o descrito na literatura.

## Material e Métodos

Estudo retrospectivo transversal aprovado pelo Comitê de Ética em Pesquisa CAAE: 68507323.8.0000.5273, com os pacientes que foram submetidos a avaliação radiográfica neste hospital no período compreendido entre janeiro de 2012 e julho de 2023.

Os critérios de inclusão utilizados foram: (1) Pacientes sem osteoartrite de joelho avaliados por ortopedista. (2) Avaliação radiográfica bilateral no exame panorâmico de membros inferiores. (3) Exame radiográfico panorâmico de membros inferiores realizado previamente a qualquer procedimento cirúrgico. (4) Pacientes com idade superior a 18 anos.

Os critérios de exclusão utilizados foram: (1) Pacientes submetidos a procedimento cirúrgico de correção de deformidade prévio ao exame radiográfico em qualquer hospital. (2) Pacientes com alteração da rotação do membro inferior ou incapacidade de mensuração no momento do exame radiográfico que alterem a qualidade da avaliação (3). Presença de osteoartrite no exame radiográfico. (4) Falta de informação no prontuário físico.

Os exames radiográficos utilizados constam no banco de dados da radiologia da nossa instituição e foi utilizado o software Peekmed (PeekMed LLC, Braga, Portugal) para mensuração dos parâmetros angulares e agrupados em tabela no Microsoft Excel (Microsoft Corp., Redmond, WA, EUA), não sendo identificados por número de prontuário ou iniciais do nome, somente por código que o pesquisador principal cadastrou, para posterior avaliação estatística. No prontuário físico foram coletadas somente as variáveis idade e sexo.

A amostra total foi classificada conforme os parâmetros sugeridos por MacDessi et al., 16 ou seja, a classificação *Coronal Plane Alignment of the Knee* (CPAK). Esta classificação é dividida em nove fenótipos de acordo com a relação entre obliquidade da linha articular (somatório do ângulo medial da tíbia proximal e o ângulo lateral distal do fêmur) e a diferença aritmética entre estes ângulos. A diferença aritmética demonstra o tipo de eixo. Quando negativa, o eixo é varo; se igual a zero, o eixo é neutro; e se positiva, o eixo é valgo. A obliquidade da linha articular somente informa para onde está o ápice da obliquidade desta linha.

## Análise Estatística

As análises foram realizadas de forma descritiva para os dados quantitativos e apresentadas em forma de média e desvio padrão, assim como mediana, valor mínimo e valor máximo. As variáveis categóricas foram expressas através de suas frequências e porcentagens.

Foi utilizado o software JASP, versão 0.18 (código aberto) para comparar os parâmetros que apresentar distribuição normal sendo utilizado o teste t de Student. As variáveis categóricas foram analisadas com o teste de Qui-Quadrado ( *Chi-squared* ) com valor de *p* < 0.05 sendo considerado como significativo.

## Resultados

Foram encontrados 621 pacientes (1.242 membros inferiores) que realizaram o exame radiográfico no período compreendido entre janeiro de 2012 e julho de 2023.

Do total da amostra, 498 pacientes eram do sexo masculino (80.1%) e 123 do sexo feminino (19.9%). A média de idade da amostra foi de 33.4 anos (desvio-padrão = 10.1). Quando comparados os desvios do eixo mecânico, sem avaliação do segmento onde encontrava a deformidade, com relação a frequência absoluta, sexo e idade; o tipo de eixo neutro foi observado em 875 membros (686 membros em indivíduos do sexo masculino e 189 membros do sexo feminino) com idade média de 32.9 anos, variando de 18 a 67 anos. No grupo que apresentou desvio do eixo em valgo ou varo houve semelhança quanto a idade média e variação. Apresentando média de idade de 34.9 anos, com variação de 18 a 66 anos em 367 membros inferiores ao total (310 membros de indivíduos no sexo masculino e 57 membros no sexo feminino) ( Tabela 1 ).

**Tabela 1:** Medidas descritivas quando comparados os desvios do eixo mecânico

	Grupo	N	Média	Mediana	Desvio-padrão	Valor de *p*
**Idade (anos)**	Neutro	875	32.92	32.00	9.956	0.004
	Valgo/Varo	367	34.75	34.00	10.65	
**NSA (graus)**	Neutro	875	126.94	127.00	5.845	< 0.001
	Valgo/Varo	367	128.78	128.00	5.24	
**LPFA (graus)**	Neutro	875	90.57	90.00	4.639	< 0.001
	Valgo/Varo	367	92.49	91.00	6.55	
**LDFA (graus)**	Neutro	875	86.72	87.00	1.530	0.176
	Valgo/Varo	367	86.52	86.00	3.75	
**JCLA (graus)**	Neutro	875	1.32	1.20	0.685	< 0.001
	Valgo/Varo	367	1.76	1.50	1.30	
**Tamanho da obliquidade da linha articular (graus)**	Neutro	875	1.44	1.10	1.062	< 0.001
	Valgo/Varo	367	2.23	1.80	1.86	
**MPTA (graus)**	Neutro	875	87.16	87.00	1.588	< 0.001
	Valgo/Varo	367	84.27	83.00	4.20	
**LDTA (graus)**	Neutro	875	88.04	88.00	1.458	< 0.001
	Valgo/Varo	367	89.04	89.00	4.49	

Abreviaturas: JCLA, *joint line convergence angle* ; LDFA, lateral distal femoral angle; LDTA, *lateral distal tibia angle* ; LPFA, *lateral proximal femoral angle* ; MPTA, *medial proximal tibia angle* ; NSA, *neck shaft angle* .

Nota: Teste T de Student.

Na avaliação dos pacientes que apresentavam desvio do eixo mecânico dos membros inferiores, sem relacionar em qual segmento se encontrava a deformidade, foi observado a predominância do desvio em varo em detrimento ao desvio em valgo em 84.4%. Houve a presença de desvio bilateral em 108 pacientes (216 membros) nos casos de varo e 15 pacientes (30 membros) dos casos de valgo. O sexo masculino foi mais frequente no varo em 56.4% dos casos, entretanto, no valgo 50.8% dos pacientes eram do sexo feminino ( Tabela 2 ).

**Tabela 2:** Padrões angulares dos membros que apresentaram desvio do eixo mecânico

	Tipo de Eixo	N	Média	Mediana	Desvio-padrão	Mínimo	Máximo	Valor de *p*
Idade (anos)	Varo	310	35.55	32	10.14	18	67	< 0.001
	Valgo	57	31.48	32	10.12	18	56	
NSA (graus)	Varo	310	128.83	128.50	5.23	115	143	0.686
	Valgo	57	128.53	127	5.30	120	150	
LPFA (graus)	Varo	310	92.56	91.00	6.50	74	115	0.604
	Valgo	57	92.07	92	6.88	70	108	
LDTA (graus)	Varo	310	87.15	87.00	3.08	73	107	< 0.001
	Valgo	57	83.11	85	5.05	72	97	
JCLA (graus)	Varo	310	1.72	1.50	1.22	0.100	8.30	0.183
	Valgo	57	1.97	1.50	1.69	0.100	7.20	
Obliquidade da Linha Articular (graus)	Varo	310	2.03	1.70	1.36	0.200	9.00	< 0.001
	Valgo	57	3.29	2.00	3.32	0.200	19.00	
MPTA (graus)	Varo	310	83.08	83.00	2.65	75	95	< 0.001
	Valgo	57	90.77	92	5.08	81	115	

Abreviaturas: JCLA, *joint convergence line angle* ; LDFA, *lateral distal femoral angle;* LDTA, *lateral distal tibial angle* ; MPFA, *medial proximal femoral angle;* MPTA, *mechanical proximal tibial angle* ; NSA, *neck shaft angle* .

Nota: Teste T de Student.

Nos pacientes com etiologia não traumática do desvio do eixo mecânico, o varo foi mais frequente nas deformidades isoladas no segmento tibial em 253 membros. No segmento femoral a deformidade isolada foi observada de 10 membros. Contudo, houve concomitância de deformidades nos segmentos tibial e femoral foi em 17 membros.

Na origem traumática, assim como na etiologia anterior, houve predomínio do varo isolado no segmento tibial em 10 membros, isolado no segmento femoral em 8 e concomitância de varo nos segmentos em 2 membros. Em 10 membros houve a concomitância de deformidade com etiologias diferentes em segmentos diferentes.

Quanto a localização do ápice da deformidade, na etiologia não traumática a localização mais frequente foi no terço proximal na deformidade em varo da tíbia. Enquanto a maior frequência observada na etiologia traumática foi no terço médio na deformidade em varo do fêmur.

No desvio em valgo do eixo mecânico foi observado a prevalência da deformidade isolada na tíbia em ambas as etiologias (7 membros na etiologia traumática e 32 membros na etiologia não traumática). No fêmur, isoladamente, a etiologia não traumática apresentou 7 casos e a traumática 1 caso. Nos casos em que a deformidade era em ambos os segmentos, fêmur e tíbia, foi observado 10 casos na etiologia não traumática. A localização da deformidade foi predominante no terço médio da tíbia na etiologia não traumática e traumática ( Tabela 3 ).

**Tabela 3:** Descrição do tipo de desvio do eixo mecânico de acordo com a etiologia, localização e variação angular da amostra

Tipo de eixo	Etiologia	Localização da deformidade	Tamanho da deformidade (graus) (mínimo–máximo; média)
**Varo**	**Não traumática**	Tíbia médio	6–14; 9
		Tíbia proximal	2–14; 8
		Fêmur médio	4–22; 12
		Fêmur distal	3–30; 9
	**Traumática**	Tíbia médio	8–20; 9
		Tíbia distal	7–21; 14
		Tíbia proximal	7–20; 13
**Valgo**	**Não traumática**	Fêmur distal	3–20 ; 8
		Fêmur médio	26
		Tíbia proximal	3–21; 7
		Tíbia médio	3–10; 6
		Tíbia distal	4–19, 12
	**Traumática**	Fêmur médio	5
		Tíbia médio	13–26; 19

Na região anatômica do joelho além dos ângulos articulares relacionados ao eixo mecânico também podem ser traçados ângulos de relação entre as superfícies articulares dos côndilos femorais e platôs tibiais ( *JCLA – Joint Convergence Line Articular* ) assim como a relação entre esta linha articular dos platôs tibiais com a linha horizontal do solo (tipo de obliquidade da linha articular dos platôs tibiais).

A amostra com deformidade em valgo do eixo mecânico do membro apresentou proporcionalmente mais casos de alteração do JCLA e relação da faixa de obliquidade frente à amostra com deformidade em varo ( Tabela 4 ).

**Tabela 4:** Variações dos parâmetros angulares *joint line convergence angle* (JCLA), tipo de obliquidade e faixa de obliquidade do joelho na amostra

Faixa JLCA	Valgo	Varo	Neutro
0 **–** 2	35	223	796
2.1 **–** 5	20	81	78
> 5.1	3	5	1
**Tipo de obliquidade**			
**Inclinação lateral** **(IL)**	26	259	675
**Inclinação medial (IM)**	32	50	200
**Faixa de obliquidade**			
0 **–** 2	30	185	726
2.1 **–** 4	13	104	122
4.1 **–** 6	8	15	23
6.1 **–** 8	1	4	3
> 8	6	1	1

A amostra total e os respectivos grupos foram classificados conforme os parâmetros sugeridos por MacDessi et al., 16 ou seja, a classificação CPAK. Na amostra total foi observado que o tipo I da classificação CPAK foi a mais frequente (44.8%) seguido pelo tipo III (37.1%). A amostra em varo seguiu o mesmo padrão da amostra geral, entretanto na amostra em valgo o tipo III foi mais frequente do que o tipo I ( Fig. 2 ).

**Fig. 2:** Classificação da amostra conforme a classificação CPAK.

## Discussão

As deformidades devem ser corrigidas devido ao aumento da pressão dos compartimentos do joelho, que gera alteração da homeostasia dos condrócitos e acelera o processo de osteoartrite. 19 20 21 22 Com o eixo dito neutro, o peso do indivíduo gera sobrecarga no compartimento medial em até 60%. Nos desvios laterais do eixo mecânico, ocorre o desvio do eixo mecânico em valgo, e a cada grau de desvio lateral da força peso, há o aumento da chance de osteoartrose unicompartimental em até 55% e 19% de osteoartrose grave nos 3 compartimentos do joelho. 20 23 Contudo, não há somente desgaste articular precoce no joelho. Aumento da sobrecarga ocorre nas articulações do tornozelo e complexo articular do pé, gerando alterações compensatórias para que o indivíduo possa realizar marcha com o menor gasto energético e articular. 24 25 O eixo mecânico neutro, sem a presença de deformidades segmentares, apresenta o paralelismo das articulações do membro inferior em relação ao solo.

Na literatura atual não há classificação que relacione etiologia com o segmento acometido e demonstre os padrões mais comuns em cada forma de deformidade. Mullaji et al. 18 descreveram uma classificação para fenótipos de pacientes apresentando a deformidade em valgo do membro inferior com objetivo de guiar o balanço de partes moles no intraoperatório de artroplastia total do joelho, não havendo menção sobre outras deformidades e etiologia. Já MacDessi et al. 16 apresentaram outra forma de classificação baseada na morfologia da linha articular do platô tibial e o tipo de desvio do eixo mecânico, não relatando o local do CORA e sua magnitude.

Em nosso estudo foi encontrado padrão de predominância de desvio em varo do eixo mecânico assim comorelatado por Moon et al. 26 e Krajnc e Drobnič. 17 Entretanto, existem diferenças entre os estudos, como a média de idade, que no nosso estudo apresentou pacientes mais jovens do que foi citado por Moon et al. (64.6 ± 13 anos). Moon et al. utilizaram 94 radiografias panorâmicas de membros inferiores com pacientes que poderiam apresentar osteoartrite de joelho. Processos degenerativos articulares do joelho podem superestimar os valores dos parâmetros angulares. Adicionalmente, há ausência de parâmetros angulares discriminados nos dados no trabalho de Moon et al. inviabilizando a comparação de forma completa. 26 Já Krajnc e Drobnič. 17 avaliaram os parâmetros angulares de 48 jogadores profissionais de futebol apresentando média da idade menor que a do nosso estudo (24.2 ± 3.6 anos) com presença de 87.5% com desvio em varo do eixo mecânico dos membros inferiores. Na comparação da amostra atendida em um hospital com indivíduos esportistas profissionais houve semelhança nos parâmetros angulares propostos por Paley et al. 8 assim como nossos resultados apresentados. Apesar da similaridade dos parâmetros angulares e tipo de desvio do eixo mecânico com os do nosso estudo, não há citação sobre a obliquidade da linha articular do joelho, JCLA e NSA.

A classificação do tipo de eixo mecânico do membro inferior por radiografia panorâmica dos membros inferiores é de fácil avaliação; em situação ideal, há a colinearidade entre o centro da cabeça do fêmur, do joelho e do tornozelo. As articulações dos membros inferiores e suas linhas de referência articulares devem ser paralelas entre si e com o solo e com o eixo passando no centro do joelho e do tornozelo (teoria do paralelismo articular). Contudo, é complexa a avaliação segmentar devido à possibilidade de deformidades complexas em um ou ambos os segmentos que somados resultam no desvio do eixo mecânico. O fenótipo populacional brasileiro é diverso e de difícil padronização. O padrão apresentado pela amostra de pacientes deste estudo difere da amostra de pacientes sem osteoartrite avaliada por MacDessi et al. 16 MacDessi et al. avaliaram 2 grupos de pacientes australianos, sendo um grupo de pessoas saudáveis sem queixas ortopédicas com idade entre 20 e 27 anos no grupo sem osteoartrite de joelho, e os classificou com relação ao eixo e tipo de obliquidade da linha articular do joelho (classificação CPAK). O tipo II da classificação CPAK foi o mais frequentemente observado seguido pelo tipo I, diferentemente do que foi observado em nossos resultados. 16 No nosso estudo foram utilizados exames radiográficos de pacientes que apresentavam algum tipo de queixa ortopédica, fato este que levou à necessidade do exame; portanto, isso pode explicar a diferença entre o padrão do nosso estudo e a amostra australiana, além da diferença de fenotipagem populacional. Já Toyooka et al. 27 utilizaram a mesma classificação para comparação de uma amostra da população japonesa que apresentavam osteoartrite, com resultados semelhantes aos do nosso estudo, com a maioria classificada como tipo I, em 53.7% da amostra total do seu estudo.

Na obliquidade da linha do joelho e JCLA foi observada a predominância dentro dos padrões da normalidade, assim como Kubota et al. 28 publicaram em seu estudo. Esses autores mantiveram o seguimento de 68 participantes com avaliação pré e pós-operatória após osteotomia valgizante da tíbia proximal por desvio em varo do eixo mecânico em pacientes com osteoartrite do compartimento medial do joelho. Na avaliação pré-operatória a obliquidade da linha articular do joelho foi de 1.1 ± .3 graus e da JCLA de 3.4 ± 2.2 graus, não identificando o CORA ou tamanho da deformidade. A importância destes parâmetros articulares é o risco de desenvolvimento precoce de osteoartrite de joelho, assim como Song et al. 29 citaram em seu trabalho. Esses autores avaliaram 109 pacientes submetidos à osteotomia de abertura medial na tíbia entre os anos de 2010 e 2015, com seguimento médio de 55 meses para avaliação da obliquidade da linha do joelho. Quando essa angulação é superior a 6 graus, há relação positiva com osteoartrose de joelho observada no exame radiográfico; e quando maior que 4 graus, há aumento de desfecho clínico negativo.

No estudo de Bagaria et al., 30 com uma amostra de 2.279 pacientes com osteoartrose de joelho analisados de 1990 a 2019 por radiografia panorâmica de membros inferiores, é citada a maior frequência de fenótipos com desvio em varo do eixo mecânico (38.7%) seguido pelo neutro (37%). Entretanto, o fenótipo mais frequente foi o varo femoral com a tíbia neutra. Nosso estudo apresentou resultados semelhantes quanto ao tipo de desvio encontrado na população; entretanto, diferentemente do seu achado, o eixo neutro foi o mais observado e, quando apresentado desvio, o varo foi mais frequente que o valgo; além disso, a deformidade em varo foi mais frequente na tíbia do que no fêmur.

Este estudo apresentou limitações quanto à definição da amostra, pois foram avaliados somente pacientes com idade superior a 18 anos sem osteoartrose. Na avaliação radiográfica, houve perda significativa de participantes devido ao mal de posicionamento para realização da radiografia panorâmica de membros inferiores. A comparação com outros estudos é dificultada por não haver estudos descritivos com todos os parâmetros angulares, somente estudos específicos de cada parâmetro, assim como ausência de estudos com amostra populacional brasileira. A miscigenação da população brasileira pode levar a diferentes fenótipos nas diferentes regiões do país, o que poderia levar a resultados diferentes do que foi encontrado. São necessários mais estudos com amostras maiores e em hospitais que não de referência em ortopedia para a comparação com os resultados apresentados neste estudo, assim como o descrito na literatura.

## Conclusão

Foram identificadas alterações nos parâmetros angulares apresentadas pela amostra de pacientes de um hospital de referência do SUS com características diferentes de amostras populacionais de outros países.
